# Sensitivity Improvement of a Humidity Sensor Based on Silica Nanospheres on a Long-Period Fiber Grating

**DOI:** 10.3390/s90100519

**Published:** 2009-01-16

**Authors:** Diana Viegas, Javier Goicoechea, José Luís Santos, Francisco Moita Araújo, Luís Alberto Ferreira, Francisco J. Arregui, Ignacio R. Matias

**Affiliations:** 1 Faculdade de Ciências da Universidade do Porto, Rua do Campo Alegre, 687, 4169-007 Porto, Portugal; 2 INESC Porto, Rua Dr. Roberto Frias, 378, 4200-465 Porto, Portugal; E-Mails: jlsantos@inescporto.pt (J. L.S.); faraujo@inescporto.pt (F. M. A.); laaf@inescporto.pt (L.A.F.); 3 Universidad Pública de Navarra, Campus Arrosadia, 31006 Pamplona, Spain; E-Mails: javier.goico@unavarra.es (J. G.); parregui@unavarra.es (F. J. A.); natxo@unavarra.es (I. R. M.)

**Keywords:** Humidity sensor, long-period fiber grating, nanospheres, electrostatic self-assembly

## Abstract

This work addresses a new configuration that improves the sensitivity of a humidity sensor based on a long-period fiber grating coated with a SiO_2_-nanospheres film. An intermediate higher refractive index overlay, deposited through Electrostatic Self-Assembly, is placed between the fiber cladding and the humidity sensitive film in order to increase the total effective refractive index of the coating. With this intermediate design, a three-fold improvement in the sensitivity was obtained. Wavelength shifts up to 15 nm against 5 nm were achieved in a humidity range from 20% to 80%.

## 1. Introduction

Fiber optic humidity sensors have been widely explored due to their practical importance in several fields in which specific environments require small sensor size and electromagnetic immunity. Several sensing fiber architectures such as hollow core fibers, tapered optical fibers, side-polished fiber, U-bends, nano-Fabry-Perot cavities etc., have been reported for humidity measurements induced by refractive index changes of the external medium [1–4]. Also, different kinds of long-period fiber gratings (LPG) [5] have been exploited as sensing transducers due to their high sensitivity to the surrounding medium.

In previous works, humidity sensors based on polymeric overlays have been developed [6–8]. However, some of them exhibit only modest performance in terms of sensitivity and time response. In the sensors proposed in the present paper an LPG is used to measure the effective refractive index changes of the polymeric overlay. In the presence of humidity, the external refractive index increases, inducing changes on the optical properties that are easily detected through a shift of the LPG resonant peak. The time response of this type of sensors as well as their sensitivity can be optimized by adding an intermediate overlay with higher refractive index, as will be further explained.

It was proved that SiO_2_ nanospheres, as a porous hydrophilic material, can be used as a humidity sensitivity film. In previous work [8] it was also demonstrated that SiO_2_ nanospheres coated on a LPG could provide a fast response humidity sensor. However, due to its low index of refraction, lower than the fiber itself, the sensitivity is quite low and should be improved in order to avoid expensive equipment. This can be done by increasing the total effective refractive index of the sensitive coating using an intermediate overlay with higher refractive index.

## 2. Theoretical Considerations

In order to analyze the spectrum evolution in structures based on LPG with a sensing overlay when the aim is only to detect the displacement of the resonance wavelengths, it is possible to use either the Bragg or the Bragg modified conditions [11]. The first one can be expressed as:

(1) $$\beta_{01}(\lambda )-\beta_{0j}(\lambda )=\frac{2\pi }{\Lambda }$$

where *β_01_* and *β_0j_* are the propagation constants of the core and the *j* cladding modes respectively, and Λ is the period of the grating. Results obtained using this approximation present appreciable variation when compared with those values calculated with rigorous coupled mode differential equations [12]. However, if the modified first-order Bragg condition is applied, errors are lower than 0.1 % [13]:

(2) $$\beta_{01}(\lambda )+s_{0}\zeta_{01,01}(\lambda )-\left(\beta_{0j}(\lambda )+s_{0}\zeta_{0j,0j}(\lambda )\right)=\frac{2\pi N}{\Lambda }$$

where *ζ_01,01_* and *ζ_0j,0j_* are the self-coupling coefficients of the core and the *j* cladding modes, *s_0_* is the coefficient of the first Fourier component of the grating and *N* is the refraction order. If the grating function is sinusoidal, *s_0_* simplifies to unity. In the case of arc-induced LPG, this last approximation can be used without lacking rigor because the margin of error is compared with the fabrication tolerances of the own LPG.

The theoretical analysis of this problem [5] shows that the sensitivity of the cladding mode to changes of the external refractive index increases when this index approaches that of the cladding. In the large majority of applications there is no opportunity for modifying this refractive index, since it is the measurement target. Therefore, one approach is to build up specific layers on the fibre surface, with proper refractive indexes and thicknesses that have the property of substantially enhancing the LPG spectral response to variations of the refractive index of the external medium.

**Figure 1.**
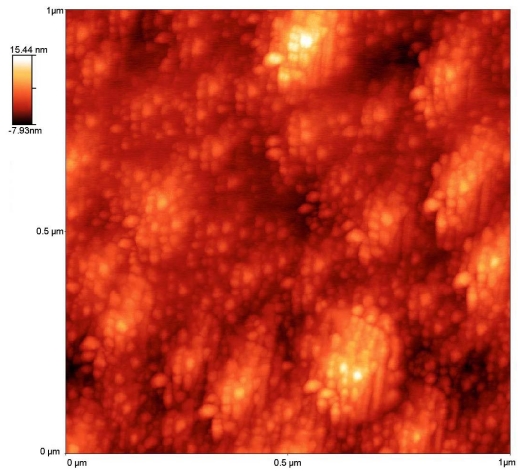
AFM image of the SiO_2_ coating.

In some theoretical works, the wavelength shift has been rigorously determined by applying a more complex theory based on a vectorial method that uses hybrid modes and coupled mode theory [14]. Using this method and a two-overlay coating based analysis, this structure can be studied theoretically. Nevertheless, if we assume only one layer with a total effective index that considers both layers, higher and lower, it is also possible to analyze and predict the behavior of the proposed sensor in a more straightforward way. The immediate consequence of the shift in effective index due to the change in the ambient humidity is a displacement in all the attenuation bands, especially in the one under study and centered at 1,540 nm. In this type of porous sensitive nanofilms, the amount of water molecules in the interstitials gaps among the nanospheres is proportional to the increase of humidity. Figure 1 shows an AFM microscope image of the SiO_2_ rough surface. The sensing mechanism is quite simple: when the ambient humidity increases, the total effective refraction index of the coating raises, increasing the value of the propagation constants of the cladding modes with respect to the propagation constant of the core mode yielding a wavelength blue shift of the resonant peak.

This maximum shift is achieved when the effective index of the mode (the resonant peak) is halfway between its minimum value and the wavelength of the next lower cladding mode before deposition. More theoretical details can be seen in [9].

## 3. Experimental

The LPG used in this study was an arc-induced LPG [10] with Λ = 395 μm and length of ∼41 mm, written on a single mode fiber (Corning SMF28). The LPG was coated with a first overlay of higher refractive index than that of the cladding (PDDA/PolyR-478) with the only purpose of increasing the total effective refractive index of the coating, and then with a sensitive-to-humidity coating of lower refractive index (PAH/SM30).

The polymeric layers were deposited using the ESA layer-by-layer method [11]. In this work the materials involved were poly(diallyidimethyl) ammonium chloride (PDDA), PolyR-478, poly(allylamine) hydrochloride (PAH) and LUDOX^®^ SM-30 SiO_2_-water colloid. In this case, the PAH and PDDA acted as polycations, and PolyR-478 and SM-30 were the anionic species. The number of overlays of PDDA/PolyR-478 was 14 layers and the number of PAH/SM30 overlays was 14 layers, which confer a total film thickness of less than 300 nanometers. Specifically, although the film thickness is difficult to measure due to the fibre geometry, in previous works it has been demonstrated that each PDDA/PolyR-478 and PAH/SM30 layers had thicknesses of approximately 12 nm and 7 nm, respectively [1, 7].

**Figure 2.**
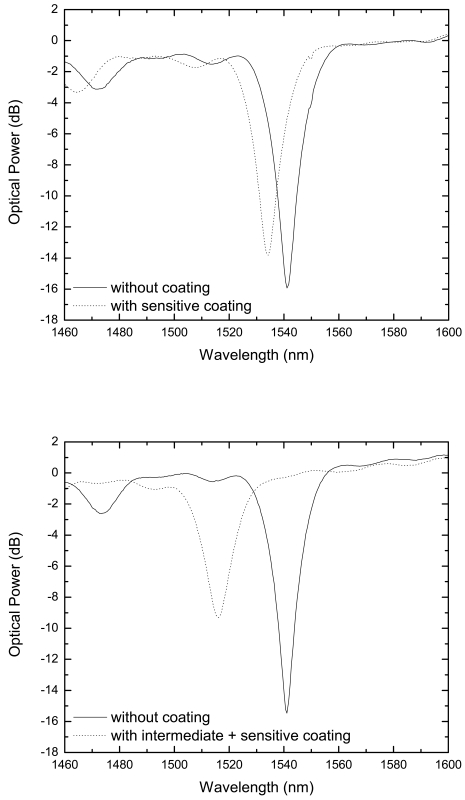
a) LPG spectra before and after deposition of PAH/SM30 (only sensitive layer), b) LPG spectra before and after deposition of PDDA/PolyR-478 + PAH/SM30 (with the intermediate layer).

The thickness overlay is chosen to guarantee that the attenuation band is located where there is good sensitivity and where it does not vanish at the same time. In this work, the good sensitivity band corresponds to ∼1,520 nm. In fact, the same effect noticeable when the ambient humidity increases, can be appreciated when the thickness of the coating is getting increased (see Figures 2a and 2b). If the sensor is located near the vanishing area (around 1,500 nm using this LPG), it would be more sensitive to any humidity change, but it would not be possible to detect any change once the peak vanished. On the other hand, if the sensor is placed near the minimum resonant peak, the humidity sensitivity would be almost negligible, because the resonant peak shift would be extremely slow, as experimentally demonstrated in [8]. A halfway decision is more convenient to work out both design features. It is important to stop the building process of the sensitive layer at a point halfway between the minimum value and the value where the resonant peak vanishes. So, there must be a trade-off between sensor sensitivity (far from the minimum because the blue shift dependence is higher) and sensor maximum wavelength displacement detection range. Using the intermediate layer, the wavelength working point can be easily located in a more favorable value.

**Figure 3.**
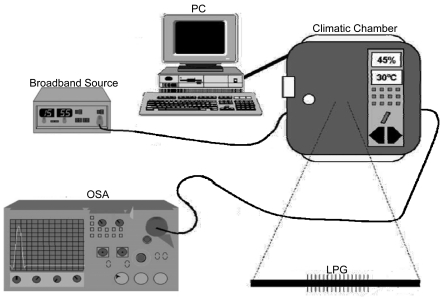
Experimental Setup.

**Figure 4.**
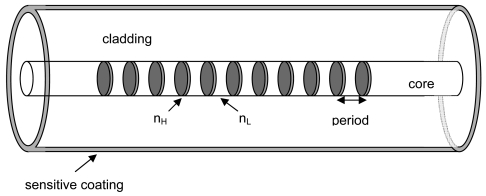
ESA coating onto a LPG [15].

In Figure 3 the experimental setup used to do the humidity tests is shown. The LPG was placed inside a climatic chamber, with temperature and humidity control, and was illuminated using a broad band source. The transmission spectrum was detected and captured by an optical spectrum analyzer. The humidity tests were made at a constant temperature of 25°C. The LPG was coated with the ESA overlay as shown in Figure 4.

## 4. Results and Discussion

**Figure 5.**
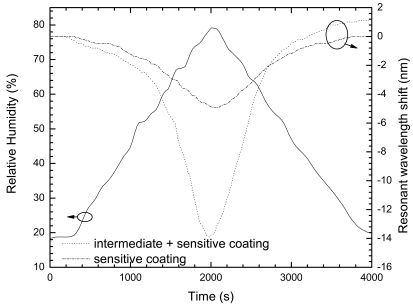
Behavior of the resonant wavelength shift for both coatings with humidity changes.

**Figure 6.**
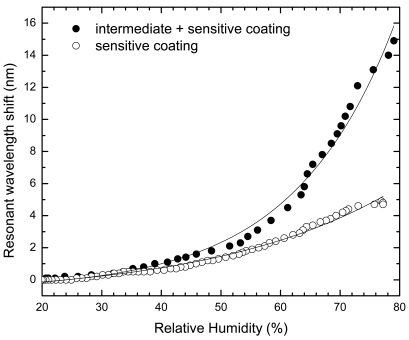
Resonant wavelength shift dependence with relative humidity for both coatings.

Figure 5 shows the resonant wavelength shift of the LPG, at room temperature, with both films for the same humidity cycle, with a range from 20% to 80%. As can be noticed, there is an obvious raise of the sensitivity for the same humidity level, when comparing both sensor head designs. The dependence of the absolute value of the resonance wavelength shift on the relative humidity can be plotted, as given in Figure 6. This dependence can be traduced through the following empirical exponential growth (*R_H_* in percentual values):

**Table:** 

Sensitive coating (PAH/SM30)	$\Delta \lambda =4.79\times 10^{-1}\cdot \exp\left(\frac{R_{H}}{3.00\times 10^{1}}\right)-10.69\times 10^{-1}$
Intermediate + sensitive coating (PDDA/PolyR + PAH/SM30)	$\Delta \lambda =1.57\times 10^{-1}\cdot \exp\left(\frac{R_{H}}{1.70\times 10^{1}}\right)-6.58\times 10^{-1}$

where *R_H_* is the Relative Humidity and *Δλ* the wavelength shift. These relations allow obtaining the sensing heads sensitivity at different relative humidity levels. For example, in the range 20–50%, the sensitivity is ≈ 0.04 nm/%R_H_ and 0.06 nm/%R_H_ for the PAH/SM30 coating and the PDDA/PolyR + PAH/SM30 structures, respectively, while around a relative humidity level of 70% those values are improved to ≈ 0.13 nm/%*R_H_* and ≈ 0.48 nm/%*R_H_* for the PAH/SM30 coating and the PDDA/PolyR + PAH/SM30 structures, respectively. These results indicate that the configuration using an intermediate overlay of higher refractive index improves the sensitivity of the sensor by a factor of 1.5 at a *R_H_* ≈ 30%, which is improved to a value of 3.5 when dealing with *R_H_* around 70%.

Preliminary experiments performed indicate a *R_H_* time response of the sensing head in the range 100–200 ms, far better than what is achieved using standard electrical humidity sensors

## 5. Conclusions

In summary, a new configuration that improves the sensitivity of a humidity sensor based on a LPG coated with a SiO_2_-nanospheres thin film was experimentally demonstrated. An intermediate higher refractive index overlay is placed between the fiber cladding and the humidity sensitive SiO_2_-nanospheres film in order to increase the total effective refractive index of the coating. In this way, compared with the situation of using only the layer sensitive to humidity, a sensitivity improvement factor larger than three was obtained. This result indicates a high potential of utilization of this sensing structure in the context of chemical and biochemical applications.
